# An overview of systematic reviews of acupuncture for Parkinson’s disease

**DOI:** 10.3389/fnins.2024.1415008

**Published:** 2024-08-30

**Authors:** Hua Xue, Hong-xian He, Dan Wu, Wen-hui Fan, Ya-xin Li

**Affiliations:** ^1^Department of Neurology, Sichuan Taikang Hospital, Chengdu, China; ^2^Department of Rehabilitation, Sichuan Taikang Hospital, Chengdu, China; ^3^Department of Nephrology, Sichuan Taikang Hospital, Chengdu, China

**Keywords:** acupuncture, systematic review, GRADE, Parkinson’s disease, AMSTAR 2

## Abstract

**Background:**

Many systematic reviews (SRs) have reported the efficacy of acupuncture in improving Parkinson’s disease (PD), but the quality of evidence is unknown. Therefore, it is necessary to comprehensively summarize and objectively evaluate the evidence of acupuncture for PD.

**Methods:**

Seven databases were searched to retrieve SRs on the acupuncture for PD. Two reviewers independently completed literature retrieval, screening, and data extraction. The methodological quality, risk of bias (RoB), evidence quality of the included SRs were assessed by the Assessing the Methodological Quality of Systematic Reviews 2 (AMSTAR 2), the Risk of Bias in Systematic Reviews (ROBIS), the Grading of Recommendations Assessment, Development and Evaluation (GRADE) tool.

**Results:**

A total of 24 SRs were included. According to AMSTAR 2, 6 (25%) were rated as high quality, 6 (25%) were rated as moderate quality, and 12 (50%) were rated as very low quality. The application of the ROBIS tool showed that 12 (25%) SRs were at low risk of bias. The results of GRADE showed that 8 (7.62%) outcomes provided high quality evidence, 23 (21.9%) outcomes provided moderate quality evidence, 42 (40%) outcomes provided low quality evidence, and 32 (30.48%) outcomes provided very low quality evidence.

**Conclusion:**

The overview indicates that acupuncture shows promise as a treatment for PD, although the evidence is limited and inconclusive due to methodological flaws and the heterogeneity of existing studies. Future research should focus on fully reporting methodological details and following review guidelines to produce more reliable and consistent evidence on the effectiveness of acupuncture for PD.

**Systematic review registration:**

https://inplasy.com, identifier INPLASY202480049.

## 1 Introduction

Parkinson’ s disease (PD) is the second most common neurodegenerative disease globally, with its prevalence steadily rising as the population ages (Simon et al., 2020). In 2019, approximately 8.51 million individuals worldwide were affected by PD, a number projected to double to over 12 million by 2040 due to global aging trends (Huang et al., 2023; Dorsey et al., 2018). Motor symptoms like resting tremor, abnormal posture and gait, myotonia, and bradykinesia are prevalent in PD patients. Additionally, non-motor symptoms such as cognitive impairment, affective disorders, sleep disturbances, pain, and autonomic nervous system dysfunction are also common (Armstrong and Okun, 2020). PD not only impacts patients’ self-care abilities but also imposes a significant financial burden on families and society. Pathologically, PD is characterized by the progressive degeneration of dopaminergic neurons in the substantia nigra and the formation of Lewy bodies. Biochemically, there is a decrease in dopamine (DA) transmission in the striatum and an imbalance between DA and acetylcholine (ACh) transmitters (Simon et al., 2020). Presently, drug therapy is the primary clinical approach for treating PD, with commonly used medications including compound levodopa, dopamine receptor agonists, and monoamine oxidase inhibitors (Angelopoulou et al., 2023). Although these drugs are effective in delaying the development of PD, most patients require long-term or even lifelong treatment. However, long-term use drugs can cause many adverse reactions, such as gastrointestinal discomfort, dyskinesia, mental disorders, etc., affecting clinical efficacy and reduce patients’ quality of life. Consequently, there is a growing interest in non-pharmacological interventions for PD (Olanow et al., 2009).

Acupuncture, a traditional Chinese treatment known for its simplicity and acceptance, has been utilized in the treatment of neurodegenerative diseases such as Alzheimer’s disease and Parkinson’s disease. According to the World Health Organization survey on complementary and alternative therapies, acupuncture is practiced in 113 out of 133 countries (Deuel and Seeberger, 2020; World Health Organization, 2019). A multicenter randomized controlled trial (RCT) demonstrated that combining acupuncture with conventional PD drug treatment can significantly enhance the motor function of PD patients (Li et al., 2023). Animal studies have further validated the therapeutic potential of acupuncture for PD, indicating that acupuncture at GB34 and LR3 acupoints can stimulate motor function recovery and activate dopaminergic neurons in specific brain regions via the Akt-BDNF pathway and autophagy, ultimately reducing dopaminergic neuron degeneration (Hsu et al., 2020). These findings suggest that acupuncture may have a promising role in managing the motor symptoms of PD by modulating neuronal activity in targeted brain regions. In addition, non-motor symptoms of PD patients, such as sleep disorders, constipation and fatigue have attracted increasing attention (Armstrong and Okun, 2020). These non-motor symptoms seriously affect the quality of life of patients. Constipation is the highest incidence of non-motor symptoms in PD patients, and more than 80% of PD patients have constipation symptoms. Studies have shown that acupuncture of specific brain regions and spinal cord segments can balance the activities of the sympathetic and parasympathetic nervous systems, thereby regulating the function of the brain-gut axis and improving gastrointestinal motility (Deuel and Seeberger, 2020; Zhang et al., 2023). Furthermore, between 60 and 90% of PD patients may experience sleep disorders. A recent RCT demonstrated that acupuncture can improve Parkinson’s Disease Sleep Scale (PDSS) scores, thereby enhancing sleep quality in PD patients (Yan et al., 2024). These findings suggest that acupuncture may also alleviate non-motor symptoms associated with PD.

In recent years, numerous systematic reviews (SRs) have demonstrated that acupuncture can effectively improve both motor and non-motor symptoms in patients with PD. Systematic reviews are considered to provide high-quality and reliable information in evidence-based medicine. However, the quality of evidence in SRs is influenced by the included studies and the researchers’ grasp of methodology, leading to varying reliability of conclusions. The diverse types of acupuncture therapies and outcome indicators in the literature result in inconsistent conclusions across studies, hindering direct clinical recommendations. This review employs the Assessment Tool for Systematic Reviews 2 (AMSTAR 2), Risk of Bias for Systematic Reviews (ROBIS), and Grading of Recommendations, Assessment, Development, and Evaluation (GRADE) to comprehensively evaluate SRs on acupuncture for PD treatment (Shea et al., 2017; Whiting et al., 2016; Schünemann et al., 2008). The aim is to rigorously assess the quality of relevant SRs and objectively assess the effectiveness and safety of acupuncture for PD.

## 2 Methods

### 2.1 Search strategy

We searched the PubMed, Embase, Web of Science, The Cochrane Library, China National Knowledge Infrastructure (CNKI), Wanfang Database, and Chongqing VIP database from their inception until March 1, 2024. We used a combination of subject words and free words, including “Parkinson Disease,” “Parkinson’s Disease,” “primary parkinsonism,” “Parkinsonism,” “Meta-Analysis,” “meta analysis,” “systematic review,” “acupuncture,” “electroacupuncture,” “scalp acupuncture.” In addition, we manually searched the list of references in the included SRs. Gray literature was excluded due to resource limitations. The detailed retrieval strategy is shown in Supplementary Table S1.

### 2.2 Inclusion criteria

We included SRs based on RCT of acupuncture for patients who were diagnosed with PD according to any internationally recognized clinical guidelines, regardless of symptoms or stage of PD. The experimental group interventions included manual acupuncture, electroacupuncture, scalp acupuncture, ear acupuncture, or acupuncture combined with PD conventional treatment (e.g., Madopar, levodopa, medication, repetitive transcranial magnetic stimulation). The control group interventions was treated with any other methods except acupuncture, such as sham acupuncture, placebo, PD conventional treatment, traditional Chinese medicine, and repetitive transcranial magnetic stimulation (rTMS). Assess motor and non-motor symptoms of PD as primary outcome measures in SRs: such as efficacy rate, Unified Parkinson’s disease rating scale (UPDRS), Parkinson’ s disease sleep scale (PDSS), Pittsburgh sleep quality indexs (PSQI), Hamilton Depression Scale (HAMD), Hamilton Anxiety Scale (HAMA), Standardized Swallowing Assessment (SSA), Parkinson’ s disease questionnaire (PDQ-39), et al.

### 2.3 Exclusion criteria

We excluded SRs if they met any of the following criteria: (a) The intervention was non-acupuncture based or the control group received the same acupuncture treatment as the experimental group; (b) SRs were analyzed using network meta-analysis or indirect comparison; (c)) duplicate publications; (d) unavailable full text or incomplete data; (e) other types of research, such as animal experiments, experimental protocols, conference papers.

### 2.4 Study selection and data extraction

Two reviewers (HX and YX-L) searched the databases according to a pre-established standardized search strategy. All retrieved literature was imported into the Literature Manager. Two reviewers independently screened candidate literature by reading titles and abstracts according to inclusion and exclusion criteria. The full text was then downloaded for further screening. At the same time, references were also reviewed to identify potential references. After identifying eligible studies, two reviewers independently extracted relevant data such as authors, year of publication, number of studies, sample size, interventions, outcomes, adverse effects, and conclusions. The two reviewers cross-checked the extracts and if there were discrepancies, a third reviewer (HX-H) was consulted to resolve the discrepancies.

### 2.5 Quality assessment

The evaluation for inclusion of SRs was conducted independently by two reviewers. Prior to the evaluation, each topic of the evaluation tool was discussed in depth to reach a consensus. At the end of the evaluation, the results were cross-checked by 2 reviewers. Disagreements were resolved by team discussion or by independent decision of the 3rd reviewer.

We used the Assessing the Methodological Quality of Systematic Reviews 2 (AMSTAR 2) to evaluate the methodological quality of the inclusion of SRs (Shea et al., 2017). The AMSTAR 2 scale consists of 16 items, each of which can be described as “Yes” and “No” and some of which can be described as “Partial yes”. Items 2, 4, 7, 9, 11, 13, and 15 are critical items and are used to critically assess the validity of an SRs. If there are no items deficiencies or only one non-critical items deficiency, the methodology is of high quality and the conclusions of the SRs are accurate and comprehensive; if there are non-critical items deficiencies but no critical items deficiencies, the methodology is of moderate quality and the conclusions of the SRs are accurate; if there is one critical items deficiency, with or without non-critical items deficiencies, the methodology is of low quality and the conclusions of the SRs are low; if there are more than one critical items deficiencies with or without non-critical items deficiencies, the methodology is of very low quality. The conclusion of SRs is inaccurate and incomplete.

We used the Risk of Bias in Systematic reviews (ROBIS) tool to assess the risk of bias (RoB) for SRs (Whiting et al., 2016). The assessment process was divided into three phases: (a) assessing relevance; (b) determining the degree of RoB in the SR process; and (c) judging RoB. The four key areas in phase 2 included study eligibility criteria, identification and selection of studies, data collection and study evaluation, and review and conclusions. Stage 3 judges the overall RoB based on the results of Stage 2, and finally categorizes the risk level as “Low risk,” “High risk,” and “Unclear risk”.

The GRADE (Grading of Recommendation, Assessment, Development, and Evaluation) tool was used to assess the quality of evidence (Schünemann et al., 2008). The quality of evidence was rated as high, moderate, low, or very low in four categories based on the presence of study limitations, inconsistency, imprecision, indirectness, or publication bias.

## 3 Results

### 3.1 Search results

**Figure 1:**
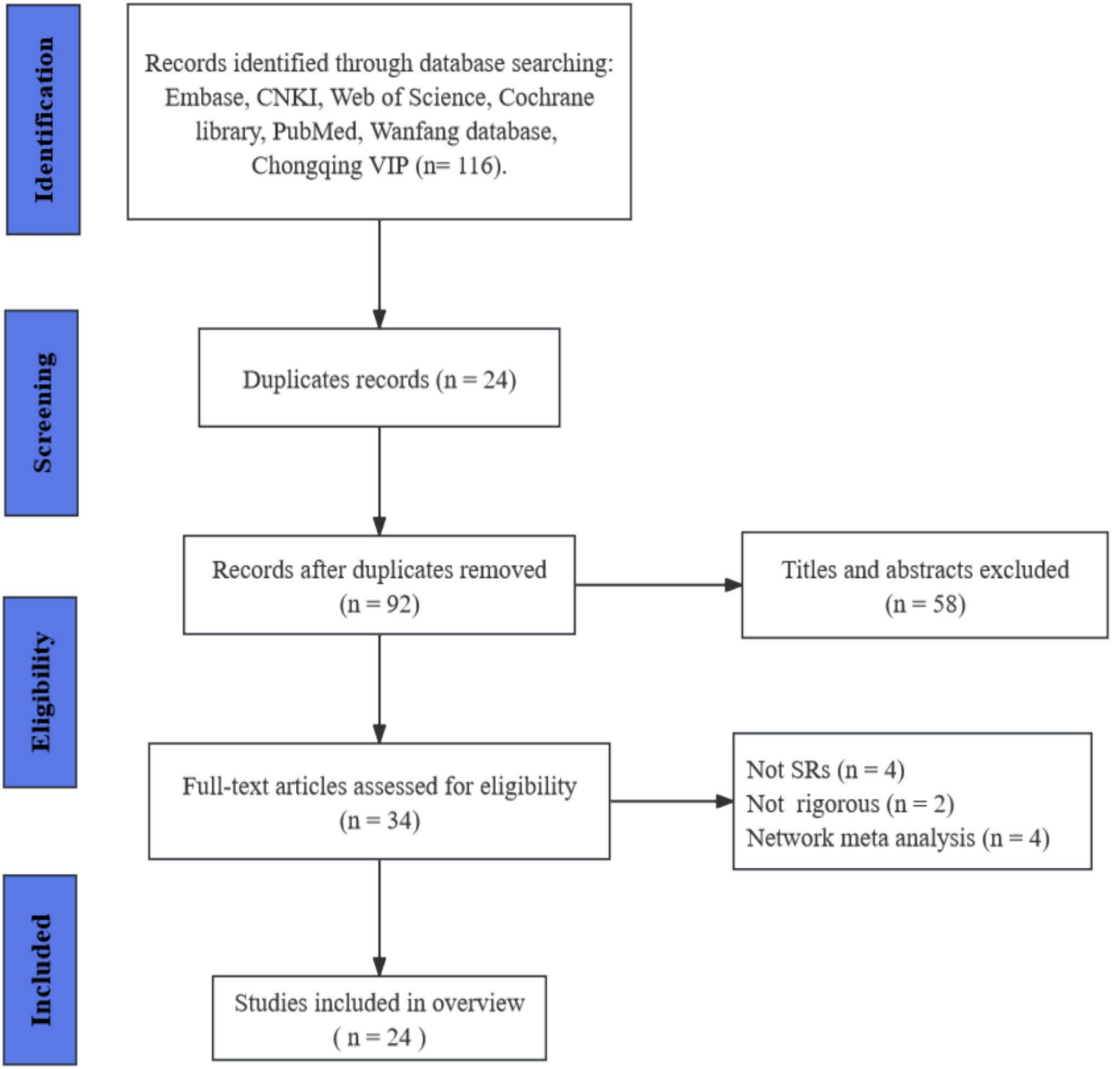
Flow chart of the literature search and study selection process.

According to the search strategy, 116 papers were retrieved, including 23 Meta-analyses and one qualitative analysis. 24 duplicates were excluded by filtration, 58 papers were screened by titles and abstracts. The remaining 34 papers were considered to be of interest. After full-text read, four papers were excluded due to not being SRs, two papers were not rigorous, four papers were network Meta-analyses. Thus, 24 papers met the inclusion criteria and were included in the final analysis (Fu and Shi, 2022; Hsu et al., 2023; Lee et al., 2008, 2013; Lee and Lim, 2017; Lei et al., 2023; Li et al., 2020, 2022; Lin et al., 2024; Liu et al., 2017, 2019; Liu and Jin, 2023; Noh et al., 2017; Ou and Xu, 2017; Sun and Zhang, 2013; Sun et al., 2023; Tan et al., 2023; Wen et al., 2021; Wu et al., 2023; Yan et al., 2024; Yang et al., 2010; Yin et al., 2016; Zhang et al., 2024; Zhou et al., 2020). The literature screening process is shown in Figure 1.

### 3.2 Characteristics of the included SRs

Table 1 presents the characteristics of the included studies. A total of 24 systematic reviews (SRs) were included that were published between 2008 and 2024, with 20 published after 2015. The number of original studies included ranged from 4 to 61, and nine SRs did not report sample sizes. Interventions in the treatment group were mainly acupuncture or acupuncture plus PD conventional treatment, rTMS, and traditional Chinese medicine, and interventions in the control group were mainly medication, rTMS, and sham acupuncture. 19 of the 24 SRs performed subgroup analyses, and eight performed sensitivity analyses. 20 SRs assessed risk of bias (RoB) using the Cochrane risk of bias tool, two used the Jadad scale, and two reviews did not mention risk of bias. In terms of conclusion, most SRs concluded that acupuncture has some advantages in treating PD, but the results still need to be validated by more and higher quality studies.

**Table 1:** Characteristics of the included systematic reviews.

Included studies	Number of studies	Participants	Experimental group	Control group	Meta analysis conducted?	Subgroup analysis conducted?	Sensitivity analysis conducted?	Risk assessment tools	Adverse effects	Outcomes	Main conclusion
Fu and Shi (2022)	13	938	Acupuncture; Acupuncture + conventional treatment; Acupuncture + traditional Chinese medicine	Sham acupuncture; conventional treatment; traditional Chinese medicine.	Yes	Yes	No	Cochrane risk of bias tool	Dizziness, upper abdominal discomfort, drowsiness, hypotension	Efficacy rate, PDSS, PSQI, UPDRS, PDQ-39	Acupuncture can significantly improve the sleep condition, related symptoms and the quality of life in Parkinson’s disease patients, and there is no difference in terms of the side effect between the experimental group and the control group
Hsu et al. (2023)	13	630	Acupuncture; Acupuncture + conventional treatment.	Sham acupuncture; conventional treatment	Yes	Yes	No	Jadad scale	Not reported	Sleep disorders, depression, anxiety and fatigue	Overall, our study highlights the potential of acupuncture as a viable complementary therapy for the treatment of PD non-motor symptoms of sleep disorders and depression, which can improve the quality of life of PD patients
Lee et al. (2008)	11	Not reported	Acupuncture; Acupuncture + conventional treatment.	Sham acupuncture; sham acupuncture + conventional treatment	No	No	No	No	Not reported	UPDRS, efficacy rate	The evidence for the effectiveness of acupuncture for treating PD is not convincing. The number and quality of trials as well as their total sample size are too low to draw any firm conclusion. Further rigorous trials are warranted
Lee et al. (2013)	4	184	Acupuncture; Acupuncture + conventional treatment	Sham acupuncture; conventional treatment	Yes	No	No	Cochrane risk of bias tool	Dull pain, gastro-intestinal upset	UPDRS, Webster scale	The result of our systematic review and meta-analysis suggested that the effectiveness of scale acupuncture for PD is promising, however, the evidence is not convincing
Lee and Lim (2017)	25	1,616	Acupuncture; Acupuncture + conventional treatment	Sham acupuncture; sham acupuncture + conventional treatment	Yes	Yes	No	Cochrane risk of bias tool	Not reported	UPDRS, Webster scales, efficacy rate	We performed a systematic review and meta-analysis to evaluate the use of acupuncture for relief of PD symptoms and found that acupuncture has significant positive effects. Acupuncture can be considered as a combination treatment with conventional treatment for patients with PD
Lei et al. (2023)	16	462	Acupuncture; Acupuncture + conventional treatment	Sham acupuncture; sham acupuncture + conventional treatment	Yes	Yes	Yes	Cochrane risk of bias tool	Dizziness, vomiting and insomnia	UPDRS III	This study found that when treating PD patients with motor symptoms, acupuncture treatment may need to reach a certain dose to obtain better therapeutic effect and excessive acupuncture stimulation may cause the body to develop a certain tolerance.
Li et al. (2020)	11	Not reported	Acupuncture; Acupuncture + conventional treatment	Conventional treatment	Yes	Yes	No	No	Not reported	UPDRS, Webster scales	Acupuncture has a significant positive effect on the clinical treatment of Parkinson ‘s disease, but for the future research of Parkinson ‘s disease, it should be explored in a more rigorous way
Li et al. (2022)	27	Not reported	Acupuncture; Acupuncture + conventional treatment	Sham acupuncture; sham acupuncture + conventional treatment	Yes	Yes	Yes	Cochrane risk of bias tool	Not reported	Insomnia, depression, cognition, constipation, fatigue, UPDRS I, UPDRS II, quality of life	The results of the analysis suggested that acupuncture treatment could ameliorate the symptoms of depression, quality of life, cognition, total mentation, behavior and mood, and activities of daily living in PD patients
Lin et al. (2024)	21	1701	Acupuncture; Acupuncture + conventional treatment; Acupuncture + rTMS	Sham acupuncture; sham acupuncture + conventional treatment; rTMS	Yes	Yes	Yes	Cochrane risk of bias tool	Not reported	PDSS, HAMA, HAMD, quality of life	This review showed that acupuncture improved sleep quality, anxiety and depressive symptoms, and quality of life of patients with Parkinson’ s disease relative to controls
Liu et al. (2017)	11	Not reported	Acupuncture; Acupuncture + conventional treatment	Sham acupuncture; sham acupuncture + conventional treatment	Yes	Yes	No	Cochrane risk of bias tool	Gastrointestinal reactions, on–off phenomena and dyskinesia, mental disorders.	Efficacy rate, UPDRS I, UPDRS II, UPDRS III, UPDRS IV	Acupuncture combined with Madopar appears, to some extent, to improve clinical effectiveness and safety in the treatment of PD, compared with Madopar alone. This conclusion must be considered cautiously, given the quality of most of the studies included was low
Liu et al. (2019)	12	864	Acupuncture; Acupuncture + conventional treatment	Conventional treatment	Yes	No	No	Cochrane risk of bias tool	Not reported	UPDRS, PDSS, Webster scales	The improvement of motor symptoms and daily living ability of PD patients treated with scalp acupuncture (or combined with western medicine) is better than that of western medicine alone, and there is no statistical significance in the improvement of sleep in PD patients
Liu and Jin (2023)	9	610	Acupuncture; Acupuncture + conventional treatment	Conventional treatment	Yes	No	No	Cochrane risk of bias tool	Not reported	Efficacy rate, SSA	Acupuncture treatment can improve swallowing function and nutrition in patients with Parkinson’s disease combined with dysphagia, but the present findings need to be validated in higher-quality studies
Noh et al. (2017)	28	2,625	Acupuncture; Acupuncture + conventional treatment	Sham acupuncture; sham acupuncture + conventional treatment	Yes	Yes	Yes	Cochrane risk of bias tool	Nausea, vomiting, constipation, and anorexia.	UPDRS, Webster scales	We found that acupuncture might be a safe and useful adjunctive treatment for patients with PD. However, because of methodological flaws in the included studies, conclusive evidence is still lacking
Ou and Xu (2017)	21	Not reported	Acupuncture; Acupuncture + conventional treatment	Sham acupuncture; sham acupuncture + conventional treatment	Yes	Yes	No	Jadad scale	Tachycardia	UPDRS, Webster scales	Acupuncture may be an effective and safe treatment for Parkinson ‘s disease
Sun and Zhang (2013)	18	1,344	Acupuncture; Acupuncture + conventional treatment	Sham acupuncture; sham acupuncture + conventional treatment	Yes	Yes	No	Cochrane risk of bias tool	Not reported	HAMD, UPDRS I, UPDRS II, Webster scales	Acupuncture treatment has a certain effect on some non-motor symptoms of PD, but it still needs more high-quality, large-sample, multi-center clinical randomized controlled trials to further confirm
Sun et al. (2023)	31	2,349	Acupuncture; Acupuncture + conventional treatment; Acupuncture + rTMS	Sham acupuncture; sham acupuncture + conventional treatment; rTMS + conventional treatment; conventional treatment	Yes	Yes	Yes	Cochrane risk of bias tool	Not reported	Efficacy rate, HAMD, MMSE, MOCA, PDSS, PSQI	The combination of acupuncture and moxibustion accompanied with other therapies is more effective than applying other therapies alone in treating non motor symptoms such as depression, cognitive impairment, sleep disturbance and constipation in Parkinson’s disease
Tan et al. (2023)	15	1,184	Acupuncture; Acupuncture + conventional treatment; Acupuncture + rTMS	Sham acupuncture; sham acupuncture + conventional treatment; rTMS + conventional treatment; conventional treatment	Yes	Yes	Yes	Cochrane risk of bias tool	Dizziness, fainting spell during acupuncture treatment	Efficacy rate, HAMD, UPDRS, UPDRS I, UPDRS II, UPDRS III, BDNF	The current evidence shows that acupuncture outperforms the control group in mitigating depression symptoms and improving daily life and motor function of PDD patients
Wen et al. (2021)	61	Not reported	Acupuncture; Acupuncture + conventional treatment	Sham acupuncture; sham acupuncture + conventional treatment	Yes	Yes	Yes	Cochrane risk of bias tool	Not reported	UPDRS, UPDRS II, UPDRS III, UPDRS I, UPDRS IV, HAMD	Acupuncture-related therapies combined with conventional medication may benefit individuals with PD. Our review findings should be considered with caution because of the methodological weaknesses in the included trials
Wu et al. (2023)	10	724	Acupuncture; Acupuncture + conventional treatment	Sham acupuncture; sham acupuncture + conventional treatment	Yes	Yes	No	Cochrane risk of bias tool	Not reported	VFSS, SSA	Acupuncture could be recommended as an adjunctive treatment for dysphagia in PD. However, due to the high risk of bias in the included studies, more high-quality evidence is needed to confirm the efficacy and safety of acupuncture for dysphagia in PD
Yan et al. (2024)	19	1,300	Acupuncture; Acupuncture + conventional treatment	Sham acupuncture; sham acupuncture + conventional treatment	Yes	Yes	No	Cochrane risk of bias tool	Not reported	PSQI, ESS, PDSS	Acupuncture therapy effectively improves nighttime sleep quality in PD patients. A treatment duration extending beyond 6 weeks is highly recommended. Additionally, increasing the frequency of acupuncture sessions and incorporating electroacupuncture in the treatment regimen may be essential for optimal results
Yang et al. (2010)	13	Not reported	Acupuncture; Acupuncture + conventional treatment	Conventional treatment	Yes	Yes	No	Cochrane risk of bias tool	Not reported	Efficacy rate, Webster scales, UPDRS	Acupuncture is safe and effective in the treatment of PD. Acupuncture plus western drugs may be superior to western drugs alone. Because of the defects in the methodological quality of the included trials, the conclusion is to be confirmed by more high quality RCTs
Yin et al. (2016)	9	665	Acupuncture; Acupuncture + conventional treatment	Conventional treatment	Yes	No	No	Cochrane risk of bias tool	Not reported	Efficacy rate	Although acupuncture may be effective for treating PD, the methodological flaws in the included studies might affect the analysis. The rigorous higher - quality RCTs are needed
Zhang et al. (2024)	13	Not reported	Acupuncture; Acupuncture + conventional treatment	Sham acupuncture; sham acupuncture + conventional treatment	Yes	Yes	Yes	Cochrane risk of bias tool	Not reported	PDSS, ESS, HAMA, HAMD, UPDRS I, UPDRS, PDQ 39	Acupuncture treatment can improve sleep quality, psychological and behavioral alterations, and the overall condition of PD patients
Zhou et al. (2020)	14	Not reported	Acupuncture; Acupuncture + conventional treatment	Sham acupuncture; sham acupuncture + conventional treatment	Yes	Yes	Yes	Cochrane risk of bias tool	Not reported	UPDRS III	Acupuncture can effectively improve the primary motor symptoms of Parkinson’s disease. The effect of acupuncture combined with western medicine is better than using western medicine only

PD, Parkinson’ s Disease; rTMS, repetitive transcranial magnetic stimulation; PDSS, Parkinson’ s disease sleep scale; PSQI, Pittsburgh sleep quality indexs; UPDRS, Unified parkinson disease rating scale; PDQ-39, Parkinson’ s disease questionnaire;ESS, Epworth Sleepiness Scale; VFSS, Videofluoroscopic Swallowing Study (VFSS) scores; SSA, Standardized Swallowing Assessment (SSA) scores; HAMD, Hamilton Depression Scale; HAMA, Hamilton Anxiety Scale.

### 3.3 Methodological assessment

The methodological quality of the 24 SRs was evaluated using the AMSTAR 2 scale. Table 2 provides an overview of the methodological quality of the included SRs. Out of the 24 SRs, 6 (25%) were rated as high quality (Hsu et al., 2023; Lei et al., 2023; Li et al., 2022; Noh et al., 2017; Wen et al., 2021; Zhang et al., 2024), 6 (25%) as moderate quality (Lee and Lim, 2017; Lin et al., 2024; Tan et al., 2023; Wu et al., 2023; Yan et al., 2024; Zhou et al., 2020), and 12 (50%) as very low quality (Fu and Shi, 2022; Lee et al., 2008, 2013; Li et al., 2020; Liu et al., 2017, 2019; Liu and Jin, 2023; Ou and Xu, 2017; Sun and Zhang, 2013; Sun et al., 2023; Yang et al., 2010; Yin et al., 2016). The compliance rate for items 1, 3, 8, 10, and 16 was 100%. Regarding critical item 2, only 10 (41.67%) SRs clearly stated the review method before conducting the review. For critical item 4, 16 (66.67%) SRs provided detailed search strategies. In terms of critical item 7, 14 (58.34%) SRs provided reasons and lists for excluded literature. The compliance rate for key items 9 and 11 was 91.67%. For critical items 13 and 15, the compliance rate was 75%, with six SRs not meeting the requirements. Among non-critical items, items 1, 3, 8, 10, and 16 have a compliance rate of 100%. When assessing non-critical items 5 and 6, study selection and data extraction were repeated for 75% of SRs. When assessing non-critical item 12, 10 (41.67%) SRs assessed the potential impact of RoB in individual studies on the results of meta-analyses or other evidence reviews. 66.67% of SRs provided satisfactory explanations and discussions of the heterogeneity observed in the review results (Table 2).

**Table 2:** Methodological quality of included systematic reviews.

Included studies	AMSTAR 2																Overall quality
	Q 1	Q 2*	Q 3	Q 4*	Q 5	Q 6	Q 7*	Q 8	Q 9*	Q 10	Q 11*	Q 12	Q 13*	Q 14	Q 15*	Q 16	
Fu and Shi (2022)	Y	Y	Y	Y	Y	Y	N	Y	Y	Y	Y	N	N	N	N	Y	Very low
Hsu et al. (2023)	Y	Y	Y	Y	Y	Y	Y	Y	Y	Y	Y	Y	Y	Y	Y	Y	High
Lee et al. (2008)	Y	N	Y	Y	Y	Y	Y	Y	N	Y	N	N	N	Y	N	Y	Very low
Lee et al. (2013)	Y	N	Y	Y	Y	Y	Y	Y	Y	Y	Y	N	N	Y	N	Y	Very low
Lee and Lim (2017)	Y	N	Y	Y	Y	Y	Y	Y	Y	Y	Y	Y	Y	Y	Y	Y	Moderate
Lei et al. (2023)	Y	Y	Y	Y	Y	Y	Y	Y	Y	Y	Y	Y	Y	Y	Y	Y	High
Li et al. (2020)	Y	N	Y	N	N	N	N	Y	N	Y	Y	N	N	N	N	Y	Very low
Li et al. (2022)	Y	Y	Y	Y	Y	Y	Y	Y	Y	Y	Y	Y	Y	Y	Y	Y	High
Lin et al. (2024)	Y	N	Y	Y	Y	Y	Y	Y	Y	Y	Y	Y	Y	Y	Y	Y	Moderate
Liu et al. (2017)	Y	Y	Y	N	N	N	N	Y	Y	Y	Y	N	N	Y	N	Y	Very low
Liu et al. (2019)	Y	N	Y	Y	Y	N	N	Y	Y	Y	Y	N	Y	Y	Y	Y	Very low
Liu and Jin (2023)	Y	N	Y	N	N	N	N	Y	Y	Y	Y	N	Y	N	Y	Y	Very low
Noh et al. (2017)	Y	Y	Y	Y	Y	Y	Y	Y	Y	Y	Y	Y	Y	Y	Y	Y	High
Ou and Xu (2017)	Y	N	Y	N	N	Y	N	Y	Y	Y	Y	N	Y	Y	Y	Y	Very low
Sun and Zhang (2013)	Y	N	Y	N	N	N	N	Y	Y	Y	Y	N	Y	N	Y	Y	Very low
Sun et al. (2023)	Y	N	Y	N	Y	Y	N	Y	Y	Y	Y	N	Y	N	Y	Y	Very low
Tan et al. (2023)	Y	N	Y	Y	Y	Y	Y	Y	Y	Y	Y	Y	Y	Y	Y	Y	Moderate
Wen et al. (2021)	Y	Y	Y	Y	Y	Y	Y	Y	Y	Y	Y	Y	Y	Y	Y	Y	High
Wu et al. (2023)	Y	Y	Y	Y	Y	Y	Y	Y	Y	Y	Y	N	Y	N	Y	Y	Moderate
Yan et al. (2024)	Y	Y	Y	Y	Y	Y	Y	Y	Y	Y	Y	N	Y	N	Y	Y	Moderate
Yang et al. (2010)	Y	N	Y	N	N	N	N	Y	Y	Y	Y	N	Y	Y	Y	Y	Very low
Yin et al. (2016)	Y	N	Y	N	Y	Y	N	Y	Y	Y	N	N	N	N	N	Y	Very low
Zhang et al. (2024)	Y	Y	Y	Y	Y	Y	Y	Y	Y	Y	Y	Y	Y	Y	Y	Y	High
Zhou et al. (2020)	Y	N	Y	Y	Y	Y	Y	Y	Y	Y	Y	Y	Y	Y	Y	Y	Moderate
Y / total %	100	41.67	100	66.67	75	75	58.34	100	91.67	100	91.67	41.67	75	66.67	75	100	

Y, yes; N, no. The key items of the AMSTAR 2. H: represents the ranking of quality as high; M: represents the ranking of quality as moderate; L: represents the ranking of quality as low; CL: represents the ranking of quality as critically low. Q1: did the research questions and inclusion criteria for the review include the components of PICO? Q2: did the report of the review contain an explicit statement that the review methods were established prior to the conduct of the review and did the report justify any significant deviations from the protocol? Q3: did the review authors explain their selection of the study designs for inclusion in the review? Q4: did the review authors use a comprehensive literature search strategy? Q5: did the review authors perform study selection in duplicate? Q6: did the review authors perform data extraction in duplicate? Q7: did the review authors provide a list of excluded studies and justify the exclusions? Q8: did the review authors describe the included studies in adequate detail? Q9: did the review authors use a satisfactory technique for assessing the risk of bias (RoB) in individual studies that were included in the review? Q10: did the review authors report on the sources of funding for the studies included in the review? Q11: if a meta-analysis was performed, did the review authors use appropriate methods for the statistical combination of results? Q12: if a meta-analysis was performed, did the review authors assess the potential impact of RoB in individual studies on the results of the meta-analysis or another evidence synthesis? Q13: did the review authors account for RoB in primary studies when interpreting/ discussing the results of the review? Q14: did the review authors provide a satisfactory explanation for, and discussion of, any heterogeneity observed in the results of the review? Q15: if they performed quantitative synthesis did the review authors carry out an adequate investigation of publication bias (small study bias) and discuss its likely impact on the results of the review? Q16: did the review authors report any potential sources of conflict of interest, including any funding they received for conducting the review? AMSTAR 2, Assessment of Multiple Systematic Reviews 2; N, no; PY, partial yes; Y, yes.

### 3.4 Results of ROBIS evaluation

The Risk of Bias in Systematic reviews (ROBIS) tool was used to assess risk of bias (RoB). All SRs included in phase 1 were rated as having a low risk of bias in terms of relevance to the research topic. In domain 1, which evaluated study eligibility criteria, 6 SRs that lacked a detailed search strategy were rated as having a high risk of bias (Li et al., 2020; Liu et al., 2017; Liu and Jin, 2023; Ou and Xu, 2017; Yang et al., 2010; Yin et al., 2016). Domain 2, which focused on the identification and selecting of studies, found 9 SRs to have a high risk of bias (Fu and Shi, 2022; Li et al., 2020; Liu et al., 2017; Liu and Jin, 2023; Ou and Xu, 2017; Sun and Zhang, 2013; Sun et al., 2023; Yang et al., 2010; Yin et al., 2016). Moving on to domain 3, which assessed the collection and appraisal of studies, 14 SRs were deemed to have a low risk of bias (Fu and Shi, 2022; Hsu et al., 2023; Lee and Lim, 2017; Lei et al., 2023; Li et al., 2022; Lin et al., 2024; Liu et al., 2017; Liu and Jin, 2023; Sun and Zhang, 2013; Sun et al., 2023; Tan et al., 2023; Zhang et al., 2024; Zhou et al., 2020). In domain 4, which examined the synthesis and findings, 14 out of the 24 SRs were rated as having a low risk of bias (Hsu et al., 2023; Lee and Lim, 2017; Lei et al., 2023; Li et al., 2022; Lin et al., 2024; Noh et al., 2017; Ou and Xu, 2017; Tan et al., 2023; Wen et al., 2021; Wu et al., 2023; Yan et al., 2024; Yang et al., 2010; Zhang et al., 2024; Zhou et al., 2020). Phase 3 evaluated the overall risk of bias of the reviews, with 12 SRs being classified as having a low risk of bias (Hsu et al., 2023; Lee and Lim, 2017; Lei et al., 2023; Li et al., 2022; Lin et al., 2024; Liu et al., 2017; Noh et al., 2017; Tan et al., 2023; Wen et al., 2021; Wu et al., 2023; Yan et al., 2024; Zhang et al., 2024; Zhou et al., 2020). For more detailed information, please refer to Table 3.

**Table 3:** Risk of bias of the included systematic reviews.

Included studies	Phase 1	Phase 2				Phase 3
	Assessing relevance	Domain 1: study eligibility criteria	Domain 2: identification and selection of studies	Domain 3: collection and study appraisal	Domain 4: synthesis and findings	Risk of bias in the review
Fu and Shi (2022)	Low risk	Low risk	High risk	Low risk	High risk	High risk
Hsu et al. (2023)	Low risk	Low risk	Low risk	Low risk	Low risk	Low risk
Lee et al. (2008)	Low risk	Low risk	Low risk	High risk	High risk	High risk
Lee et al. (2013)	Low risk	Low risk	Low risk	High risk	High risk	High risk
Lee and Lim (2017)	Low risk	Low risk	Low risk	Low risk	Low risk	Low risk
Lei et al. (2023)	Low risk	Low risk	Low risk	Low risk	Low risk	Low risk
Li et al. (2020)	Low risk	High risk	High risk	High risk	High risk	High risk
Li et al. (2022)	Low risk	Low risk	Low risk	Low risk	Low risk	Low risk
Lin et al. (2024)	Low risk	Low risk	Low risk	Low risk	Low risk	Low risk
Liu et al. (2017)	Low risk	High risk	High risk	Low risk	High risk	High risk
Liu et al. (2019)	Low risk	Low risk	Low risk	High risk	High risk	High risk
Liu and Jin (2023)	Low risk	High risk	High risk	Low risk	High risk	High risk
Noh et al. (2017)	Low risk	Low risk	Low risk	High risk	Low risk	Low risk
Ou and Xu (2017)	Low risk	High risk	High risk	High risk	Low risk	High risk
Sun and Zhang (2013)	Low risk	Low risk	High risk	Low risk	High risk	High risk
Sun et al. (2023)	Low risk	Low risk	High risk	Low risk	High risk	High risk
Tan et al. (2023)	Low risk	Low risk	Low risk	Low risk	Low risk	Low risk
Wen et al. (2021)	Low risk	Low risk	Low risk	Low risk	Low risk	Low risk
Wu et al. (2023)	Low risk	Low risk	Low risk	High risk	Low risk	Low risk
Yan et al. (2024)	Low risk	Low risk	Low risk	High risk	Low risk	Low risk
Yang et al. (2010)	Low risk	High risk	High risk	High risk	Low risk	High risk
Yin et al. (2016)	Low risk	High risk	High risk	High risk	High risk	High risk
Zhang et al. (2024)	Low risk	Low risk	Low risk	Low risk	Low risk	Low risk
Zhou et al. (2020)	Low risk	Low risk	Low risk	Low risk	Low risk	Low risk

### 3.5 Quality of evidence

The 24 SRs consisted of 105 outcomes related to the effectiveness of acupuncture in the treatment of PD with respect to effectiveness rate, UPDRS, UPDRS I, UPDRS II, UPDRS III, UPDRS IV, PDSS, PSQI, PDQ-39, ESS, Webster scale, Quality of life, HAMA, HAMD, etc. The GRADE assessment showed that 8 (7.62%) outcomes provided high quality evidence, 23 (21.9%) outcomes provided moderate quality evidence, 42 (40%) outcomes provided low quality evidence, and 32 (30.48%) outcomes provided very low quality evidence. The evidence was downgraded due to the following limitations: (1) Randomization, blinding, and allocation concealment bias in clinical studies reduce the validity of the GRADE method. (2) We downgraded the quality of the evidence based on publication bias due to incomplete literature search and the number of research clinical trials. (3) We downgrade the quality of the evidence to imprecise if the confidence interval is wide or the number of participants is small. (4) Heterogeneity was high, and we downgraded the quality of the evidence on the grounds of inconsistency. Additional details are provided in Table 4.

**Table 4:** Results of evidence quality with GRADE.

Included studies	Outcomes	Included studies	Inconsistency	Indirectness	Imprecision	Publication bias	Relative effect (95% CI)	*I* ^2^	*p*-value	Quality
Fu and Shi (2022)	Efficacy rate	−1	0	0	0	−1	RR 1.33 (1.22, 1.44)	46%	< 0.0001	Low
	PDSS	−1	−1	0	0	−1	MD 11.10 (7.51, 14.68)	78%	< 0.0001	Very low
	PSQI	−1	−1	0	−1	−1	MD 4.21 (1.41, 7.00)	97%	0.003	Very low
	UPDRS	−1	0	0	0	−1	MD −5.45 (−6.46, −4.45)	0	< 0.0001	Low
	PDQ 39	−1	0	−1	0	−1	MD −5.27 (−8.90, −1.64)	20%	0.004	Very low
Hsu et al. (2023)	Sleep disorders	−1	−1	0	−1	−1	SMD 0.549 (0.181, 0.916)	64%	0.003	Very low
	PDSS/PDSS-2	−1	−1	0	0	−1	SMD 0.695 (0.250, 1.140)	66%	0.002	Very low
	ESS	−1	0	0	0	−1	MD 2.136 (0.635, 3.637)	39%	0.005	Low
	Depression	−1	0	0	0	0	SMD 0.242 (0.055, 0.430)	0	0.011	Moderate
	Anxiety	−1	0	0	−1	−1	SMD 0.095 (−0.159, 0.348)	0	0.465	Very low
	Fatigue	−1	0	0	−1	−1	SMD 0.273 (−0.080, 0.626)	0	0.129	Very low
Lee et al. (2008)	AT *VS* SA: UPDRS	0	0	0	−1	0	WMD 5.7 (−2.8, 14.2)	0	0.19	Moderate
	UPDRS	0	0	0	−1	−1	WMD 13.56 (3.88, 23.25)	0	0.006	Low
	Efficacy rate	−1	0	−1	−1	−1	RR 1.46 (1.15, 1.87)	12%	0.002	Very low
Lee et al. (2013)	UPDRS	−1	0	0	0	0	WMD −3.94 (−6.05, −1.84)	0	0.01	Moderate
	Webster scale	−1	−1	0	0	−1	WMD 1.39 (0.79, 2.12)	84%	0.30	Very low
Lee and Lim (2017)	UPDRS I	−1	0	0	0	−1	WMD 0.27 (−0.17, 0.72)	0	0.23	Low
	UPDRS II	−1	0	0	0	−1	WMD 3.59 (2.55, 4.63)	0	< 0.001	Low
	UPDRS III	−1	0	0	0	0	WMD 4.46 (3.53, 5.39)	0	< 0.001	Moderate
	UPDRS IV	−1	0	0	0	−1	WMD 1.36 (−0.57, 3.29)	0	< 0.001	Low
	UPDRS	−1	0	0	0	0	WMD 10.37 (8.38, 10.07)	0	< 0.001	Moderate
	AT *VS* No treatment: Webster scale	−1	0	0	0	−1	WMD 7.36 (5.58, 9.14)	0	< 0.001	Low
	AT *VS* CT: Webster scale	−1	0	0	0	−1	WMD 3.08 (2.81, 3.35)	0	< 0.001	Low
	AT plus CT *VS* CT: Webster scale	−1	−1	0	0	−1	WMD 3.78 (2.17, 5.40)	93%	< 0.001	Very low
	AT plus CT *VS* CT: Efficacy rate	−1	−1	0	0	0	RR 1.35 (1.25, 1.46)	73%	< 0.001	Low
Lei et al. (2023)	UPDRS III	0	−1	0	0	0	MD −3.56 (−4.85, −2.26)	95%	< 0.001	Moderate
Li et al. (2020)	UPDRS III	−1	0	−1	−1	−1	MD −11.77 (−14.19, −9.344)	0	< 0.01	Very low
	Webster scale	−1	0	−1	−1	−1	WMD 1.106 (1.022, 1.197)	0	< 0.05	Very low
Li et al. (2022)	Insomnia	0	−1	0	−1	−1	SMD 0.064 (−0.447, 0.576)	89.5%	0.805	Very low
	AT plus CT *VS* CT: insomnia	0	−1	0	0	0	SMD 0.517 (0.242, 0.793)	47.4%	<0.0001	Moderate
	AT *VS* CT: insomnia	0	−1	0	−1	0	SMD −0.898 (−2.432, 0.636)	95.7%	0.251	Low
	Depression	0	−1	0	0	0	SMD −0.353 (−0.669, −0.037)	75.5%	0.029	Moderate
	AT plus CT *VS* CT: depression	0	−1	0	−1	−1	SMD −0.509 (−1.067, 0.049)	86.2%	0.074	Very low
	AT *VS* CT: depression	0	0	0	−1	−1	SMD −0.136 (−0.364, 0.092)	0	0.241	Low
	Cognition	0	−1	0	−1	0	SMD 0.878 (0.046, 1.711)	92.8%	0.039	Low
	AT plus CT *VS* CT: cognition	0	−1	0	−1	0	SMD 0.985 (−0.130, 2.101)	95.7%	0.083	Low
	AT *VS* CT: cognition	0	−1	0	−1	0	SMD 0.724 (−0.868, 2.316)	95.6%	0.373	Low
	Constipation	0	−1	0	0	0	SMD 0.422 (−0.201, 1.044)	65.5%	0.185	Moderate
	UPDRS I	0	−1	0	0	0	WMD −1.536 (−2.201, −0.871)	88.9%	<0.0001	Moderate
	UPDRS II	0	−1	0	0	0	WMD −2.071 (−3.792, −0.351)	83.1%	0.018	Moderate
	Quality of life	0	−1	0	0	0	SMD −0.690 (−1.226, −0.155)	89.6%	0.011	Moderate
Lin et al. (2024)	PDSS	−1	0	0	0	0	MD 10.15 (8.91, 11.38)	3%	<0.0001	Moderate
	≤4 weeks: PDSS	−1	0	0	0	0	MD 9.94 (8.60, 11.28)	0	<0.0001	Moderate
	> 4 weeks: PDSS	−1	0	0	0	0	MD 11.28 (8.14, 14.43)	31%	<0.0001	Moderate
	HAMA	−1	0	0	0	0	MD −2.46 (−3.54, −1.39)	0	<0.0001	Moderate
	HAMD	−1	−1	0	0	0	MD −1.63 (−1.97, −1.28)	43%	<0.0001	Low
	Quality of life	−1	0	0	0	0	MD −3.64 (−5.64, −1.65)	0	0.0003	Moderate
Liu et al. (2017)	Efficacy rate	−1	−1	0	0	0	RR 1.28 (1.18, 1.38)	28%	<0.001	Low
	UPDRS I	−1	−1	0	0	−1	SMD −0.37 (0.77, 0.02)	47%	0.06	Very low
	UPDRS II	−1	−1	0	0	−1	SMD −1.00 (−1.71, −0.29)	82%	0.006	Very low
	UPDRS III	−1	−1	0	0	−1	SMD −0.93 (−2.28, 0.41)	95%	0.17	Very low
	UPDRS IV	−1	−1	0	0	−1	SMD -0.78 (−2.24, 0.68)	96%	0.30	Very low
	UPDRS I - IV	−1	−1	0	0	−1	SMD −1.15 (−1.63, −0.67)	77%	<0.001	Very low
Liu et al. (2019)	UPDRS	−1	−1	0	0	−1	SMD −7.30 (−12.80, −1.79)	95%	0.009	Very low
	PDSS	−1	0	0	0	−1	SMD 2.67 (−0.27, 5.60)	0	0.08	Low
	Webster scale	−1	0	0	0	−1	SMD 2.52 (1.74, 3.64)	0	<0.0001	Low
Liu and Jin (2023)	Efficacy rate	−1	0	0	0	−1	RR 1.37 (1.24, 1.50)	0	<0.0001	Low
	SSA	−1	0	0	0	−1	MD −2.26 (−3.95, −1.29)	0	<0.0001	Low
	VFSS	−1	0	0	0	−1	MD 1.30 (0.88, 1.73)	0	<0.0001	Low
Noh et al. (2017)	UPDRS	0	−1	0	0	0	WMD −10.48 (−13.61, −7.34)	47%	<0.0001	Moderate
	UPDRS I	0	0	0	0	0	WMD −1.17 (−1.60, −0.75)	0	<0.0001	High
	UPDRS II	0	0	0	0	0	WMD −4.68 (−7.16, −2.19)	10%	0.0002	High
	UPDRS III	0	0	0	0	0	WMD −2.92 (−5.13, −0.71)	0	0.01	High
	Webster scale	0	−1	0	0	0	WMD −1.99 (−3.43, −0.56)	81%	0.006	Moderate
Ou and Xu (2017)	AT *VS* CT: UPDRS	−1	−1	0	−1	−1	WMD −2.55 (−11.15, 6.05)	70%	0.56	Very low
	AT plus CT *VS* CT: UPDRS	−1	−1	0	−1	−1	WMD −0.43 (−0.70, −0.17)	60%	0.001	Very low
	AT *VS* CT: Webster scale	−1	0	0	−1	−1	WMD −2.5 (−2.77, −2.23)	0	<0.0001	Very low
	AT plus CT *VS* CT: Webster scale	−1	0	0	−1	−1	WMD −6.48 (−20.19, 7.22)	0	<0.0001	Very low
Sun and Zhang (2013)	HAMD	−1	−1	0	0	−1	SMD −4.42 (−6.44, −2.39)	63%	<0.0001	Very low
	UPDRS I	−1	−1	0	0	−1	SMD −0.71 (−1.16, −0.26)	75%	0.002	Very low
	UPDRS II	−1	0	0	0	−1	SMD −4.24 (−5.08, −3.39)	0	<0.0001	Low
	Webster scale	−1	0	0	0	−1	OR 0.45 (0.29, 0.68)	0	0.0001	Low
Sun et al. (2023)	Efficacy rate	−1	0	0	0	−1	OR 4.07 (2.94, 5.63)	0	<0.0001	Low
	HAMD	−1	−1	0	0	−1	MD 2.27 (1.38, 3.15)	59%	0.006	Very low
	MMSE	−1	0	0	0	−1	MD −3.86 (−4.81, −2.92)	0	<0.0001	Low
	MoCA	−1	−1	0	0	−1	MD −2.80 (−3.62, −1.97)	44%	<0.0001	Very low
	PDSS	−1	−1	0	0	−1	MD −7.25 (−12.88, −1.62)	67%	0.006	Very low
	PSQI	−1	0	0	0	−1	MD 3.90 (3.35, 4.46)	0	<0.0001	Low
Tan et al. (2023)	Efficacy rate	0	−1	0	0	0	RR 1.25 (1.17, 1.33)	58%	<0.0001	Moderate
	HAMD	0	−1	0	0	0	WMD −3.38 (−4.79, −1.98)	96%	<0.0001	Moderate
	UPDRS	0	0	0	0	0	WMD −6.67 (−7.50, −5.84)	13%	<0.0001	High
	UPDRS I	0	−1	0	0	−1	WMD −1.44 (−3.02, 0.15)	95%	<0.0001	Low
	UPDRS II	0	0	0	0	0	WMD −2.19 (−2.61, −1.78)	0	<0.0001	High
	UPDRS III	0	0	0	0	0	WMD −3.32 (−3.99, −2.65)	0	<0.0001	High
	BDNF	0	−1	0	−1	0	WMD 2.47 (1.03, 3.91)	63%	<0.0001	Moderate
Wen et al. (2021)	UPDRS	−1	−1	0	0	0	MD −7.37 (−8.91, −5.82)	88%	<0.01	Low
	UPDRS II	−1	−1	0	0	0	MD −3.96 (−4.96, −2.95)	78%	<0.01	Low
	UPDRS III	−1	−1	0	0	0	MD −3.90 (−4.33, −3.47)	68%	<0.01	Low
	UPDRS I	−1	−1	0	0	0	MD −1.27 (−1.77, −0.78)	90%	<0.01	Low
	UPDRS IV	−1	−1	0	0	0	MD −1.32 (−1.87, −0.78)	89%	<0.01	Low
	HAMD	−1	−1	0	0	0	MD −2.38 (−4.64, −0.11)	93%	<0.05	Low
Wu et al. (2023)	VFSS	0	0	0	0	0	MD 1,48 (1.16, 1.81)	0	<0.0001	High
	SSA	0	0	0	0	0	MD −3.08 (−4.01, −2.15)	0	<0.0001	High
Yan et al. (2024)	PDSS	0	−1	0	0	−1	MD 10.81 (5.64, 15.98)	60%	<0.0001	Low
	PSQI	0	−1	0	0	−1	MD −4.52 (−6.36, −2.67)	94%	<0.0001	Low
	ESS	0	−1	0	0	−1	MD −0.90 (−3.67, −1.88)	78%	0.53	Low
Yin et al. (2016)	Efficacy rate	−1	−1	0	0	-1	OR 2.60 (1.78, 3.79)	18%	<0.0001	Very low
Zhang et al. (2024)	PDSS	−1	0	0	0	0	SMD 0.47 (0.19, 0.75)	18.7%	0.001	Moderate
	ESS	−1	0	0	0	-1	SMD 0.27 (−0.08, 0.62)	0	0.128	Low
	HAMA	−1	−1	0	0	-1	SMD −1.13 (−3.92, 1.67)	97.5%	0.43	Very low
	HAMD	−1	−1	0	0	-1	SMD 0.89 (−0.27, 2.04)	87.2%	0.132	Very low
	UPDRS I	−1	−1	0	0	0	SMD −0.77 (−1.31, −0.23)	60.3%	0.005	Low
	UPDRS	−1	−1	0	0	0	SMD −1.12 (−2.21, −0.04)	95.5%	0.042	Low
	PDQ 39	−1	−1	0	0	0	SMD −0.09 (−0.39, 0.21)	48.9%	0.552	Low
Zhou et al. (2020)	UPDRS III	0	−1	0	0	−1	WMD −4.13 (−5.24, −3.03)	32%	<0.0001	Low

AT, acupuncture therapy; CT, conventional therapy; PDSS, Parkinson’ s disease sleep scale; PSQI, Pittsburgh sleep quality indexs; UPDRS, Unified parkinson disease rating scale; PDQ-39, Parkinson’ s disease questionnaire; ESS, Epworth Sleepiness Scale; VFSS, Videofluoroscopic Swallowing Study (VFSS) scores; SSA, Standardized Swallowing Assessment (SSA) scores; HAMD, Hamilton Depression Scale; HAMA, Hamilton Anxiety Scale.

### 3.6 Outcome indicators and related conclusions

Ten SRs assessed the UPDRS, and eight of these SRs came to the unanimous conclusion that acupuncture in combination with conventional treatments or acupuncture treatment alone was more advantageous in improving the UPDRS than the control group (Fu and Shi, 2022; Lee et al., 2008, 2013; Lee and Lim, 2017; Liu et al., 2019; Noh et al., 2017; Ou and Xu, 2017; Tan et al., 2023; Wen et al., 2021; Zhang et al., 2024). Lee et al. (2008) SRs comparing acupuncture with placebo acupuncture resulted in acupuncture failing to be more advantageous in UPDRS (WMD = 5.7, 95% CI 22.8 to 14.2, *p* = 0.19). In addition, acupuncture combined with medication was superior to medication alone for UPDRS (WMD = 13.56, 95% CI 3.88 to 23.25, *p* = 0.006) (Lee et al., 2008). Meta-analysis by Ou et al. showed that acupuncture alone did not provide an advantage over conventional treatment for PD in terms of improvement in UPDRS between the two groups (WMD = −2.77, 95% CI −11.15 to 6.05, *p* = 0.56), and acupuncture combined with PD conventional treatment was more advantageous than conventional treatment alone (WMD = −0.43, 95% CI -0.7 to −0.17, *p* = 0.001) (Ou and Xu, 2017). Eight SRs focused on UPDRS I scores, with two SRs concluding that UPDRS I scores did not significantly improve after acupuncture treatment (Lee and Lim, 2017; Liu et al., 2017), and six SRs concluding that acupuncture therapy in combination with medications or acupuncture alone significantly improved UPDRS I scores compared with medications alone (Li et al., 2022; Noh et al., 2017; Sun and Zhang, 2013; Tan et al., 2023; Wen et al., 2021; Zhang et al., 2024). UPDRS II was the activity of daily living score, and 7 SRs focused on UPDRS II score (Lee and Lim, 2017; Li et al., 2022; Liu et al., 2017; Noh et al., 2017; Sun and Zhang, 2013; Tan et al., 2023; Wen et al., 2021). The results showed that acupuncture combined with drugs was superior to the control group in reducing UPDRS II score (Lee and Lim, 2017; Li et al., 2022; Liu et al., 2017; Noh et al., 2017; Sun and Zhang, 2013; Tan et al., 2023; Wen et al., 2021). Eight SRs focused on UPDRS III scores, with the results of one SR concluding that acupuncture therapy did not demonstrate a significant advantage over conventional treatment, and the results of seven SRs showing that acupuncture therapy was superior to controls in reducing UPDRS III scores (Lee and Lim, 2017; Lei et al., 2023; Li et al., 2020; Liu et al., 2017; Noh et al., 2017; Tan et al., 2023; Wen et al., 2021; Zhou et al., 2020). Three SRs focused on the UPDRS IV, of which two SRs considered that acupuncture combined with drug therapy had an advantage over conventional treatment in reducing the UPDRS IV score, and 1 SR considered that acupuncture combined with conventional therapy did not show a significant advantage over conventional therapy (Lee and Lim, 2017; Liu et al., 2017; Wen et al., 2021).

Seven SRs assessed the efficacy rate and showed that the acupuncture group had a higher efficacy rate than the control group (Fu and Shi, 2022; Lee et al., 2008; Lee and Lim, 2017; Liu et al., 2017; Sun et al., 2023; Tan et al., 2023; Yin et al., 2016). 7 SRs used the Webster score to evaluate the overall symptoms (Lee et al., 2013; Lee and Lim, 2017; Li et al., 2020; Liu et al., 2019; Noh et al., 2017; Ou and Xu, 2017; Sun and Zhang, 2013). 6 of them concluded that the Webster score after acupuncture treatment was lower than that after conventional treatment, and that acupuncture treatment was better than conventional treatment (Lee and Lim, 2017; Li et al., 2020; Liu et al., 2019; Noh et al., 2017; Ou and Xu, 2017; Sun and Zhang, 2013). One SR concluded that there was no significant difference in Webster scores after acupuncture treatment and conventional treatment (Lee et al., 2013). Eight SRs evaluated the efficacy of acupuncture in the treatment of sleep disorders in PD patients. The outcome indicators mainly included PDSS, PSQI, ESS (Fu and Shi, 2022; Hsu et al., 2023; Li et al., 2022; Lin et al., 2024; Liu et al., 2019; Sun et al., 2023; Yan et al., 2024; Zhang et al., 2024), of which 2 SRs considered that acupuncture had no significant effect in improving sleep disorders (Li et al., 2022; Liu et al., 2019), of which 2 SRs considered that acupuncture did not improve the ESS score (Yan et al., 2024; Zhang et al., 2024). 8 SRs focused on the mental disorder of PD patients, and outcome indicators included HAMA, HAMD, anxiety symptoms, depressive symptoms, fatigue, etc. (Hsu et al., 2023; Li et al., 2022; Lin et al., 2024; Sun and Zhang, 2013; Sun et al., 2023; Tan et al., 2023; Wen et al., 2021; Zhang et al., 2024). The results of 3 of the SRs showed that acupuncture treatment could not improve anxiety, depression, and fatigue (Hsu et al., 2023; Li et al., 2022; Zhang et al., 2024). Four articles focused on the daily living abilities of PD patients (Fu and Shi, 2022; Li et al., 2022; Lin et al., 2024; Zhang et al., 2024), and the outcome indicators included PDQ 39. One of the SR believed that acupuncture treatment did not improve the daily living abilities of PD patients (Zhang et al., 2024). Two SRs evaluated the swallowing ability of patients with PD treated with acupuncture, and the outcome indicators included SSA and VFSS (Liu and Jin, 2023; Wu et al., 2023). 2SRs demonstrated that acupuncture could improve dysphagia. Only 1 SR focused on constipation, and the results showed that acupuncture cannot improve constipation in PD patients (Li et al., 2022). Two SRs focused on the cognitive function of PD patients, and the outcome indicators included MMSE and MoCA (Li et al., 2022; Sun et al., 2023). They concluded that acupuncture can improve the cognitive function of PD.

## 4 Discussion

### 4.1 Summary of the main results

This overview provides a comprehensive descriptive analysis of 24 SRs of acupuncture for patients with PD, involving 425 clinical studies. We used AMSTAR 2, ROBIS, and GRADE to comprehensively assess the methodological quality, quality of evidence, and RoB of the published SRs. According to AMSTAR 2, 6 (25%) were rated as high quality, 6 (25%) were rated as moderate quality, and 12 (50%) were rated as very low quality. The application of the ROBIS tool showed that 12 (25%) SRs were at low risk of bias. The results of GRADE showed that 8 (7.62%) outcomes provided high quality evidence, 23 (21.9%) outcomes provided moderate quality evidence, 42 (40%) outcomes provided low quality evidence, and 32 (30.48%) outcomes provided very low quality evidence.

### 4.2 Results-based discussion

According to AMSTAR 2, 50% of systematic reviews (SRs) were deemed to have very low methodological quality, indicating a significant issue with the overall quality of SRs. Specifically, 58.33% (14/24) of SRs lacked a comprehensive plan prior to commencing the review process, raising concerns about the adherence to a structured research plan during the review. This lack of clarity in the research process can potentially introduce bias. Additionally, 8 out of 24 SRs had deficiencies in their literature search strategies due to the absence of detailed search strategies, keywords, and Mesh terms. Furthermore, 10 out of 24 SRs did not provide explanations for excluding specific literature, while 6 out of 24 SRs failed to adequately address bias in individual studies and explore publication bias. In terms of non-critical items, some SRs did not implement duplicate study screening and data extraction.

The GRADE assessment revealed that 8 (7.62%) outcomes presented high quality evidence, 23 (21.9%) outcomes presented moderate quality evidence, 42 (40%) outcomes presented low quality evidence, and 32 (30.48%) outcomes presented very low quality evidence. Study limitations, publication bias, inconsistency, and inaccuracy in systematic reviews reduce the overall quality of evidence. The majority of evidence in the original studies included in the literature was deemed to have a high risk of bias due to insufficient reporting of randomization, blinding, allocation concealment, and other factors, as well as issues such as loss of visits, withdrawals, and publication bias. To enhance the reliability of results, researchers conducting systematic reviews should thoroughly evaluate and report on these key aspects of the original literature. Some studies had small sample sizes but showed large differences in effect sizes and minimal overlap in confidence intervals, resulting in imprecise results. Furthermore, there was significant heterogeneity among the raw data, which was not adequately addressed during the analysis, thereby diminishing the quality of the evidence. Possible sources of this heterogeneity include: 1) Variations in acupuncture techniques across studies, which may involve different acupuncture points, frequencies, or treatment duration; 2) Differences in outcome measures, as studies may utilize varying scales or assess the effects of acupuncture differently; and 3) Variability in the characteristics of study participants, such as age, disease severity, and medication use. These findings highlight the importance of a thorough description of included RCTs in systematic reviews to help identify sources of heterogeneity and facilitate a more objective and comprehensive analysis of the results.

### 4.3 Mechanism of acupuncture for PD

This overview suggests that acupuncture has potential in the treatment of PD, and most of the conclusions indicate that it can alleviate the motor and non-motor symptoms of PD. The main characteristic of PD is the degeneration and loss of dopamine (DA) neurons. Dopamine neurons are located in the substantia nigra and plays an important role in regulating movement and coordinating muscle activity (Simon et al., 2020). Apoptosis may participate in the degenerative process of neurons through multiple pathways in PD, and is also one of the main pathways leading to PD (Lev et al., 2003). Increased P53 expression plays an important role in the pathogenesis of PD (Luo et al., 2022). Electroacupuncture can inhibit cell apoptosis and improve PD behavioral disorders by down regulating the P53 pathway in the striatum, providing a theoretical basis for the prevention and treatment of PD (Park et al., 2015). Acupuncture can inhibit the activation of the MAP4K3/MKK4/JNK pathway, thereby alleviating cell death and pathological changes in the hippocampal tissue of PD rats, while also improving their cognitive and behavioral functions (Park et al., 2015). Research indicates a close relationship between oxidative stress and Parkinson’s disease (PD) (Zhao et al., 2022). Acupuncture treatment has been shown to regulate oxidative stress indicators in patients by reducing malondialdehyde levels and increasing superoxide dismutase, glutathione peroxidase, and catalase levels (Zhao et al., 2022). Additionally, acupuncture has been found to regulate oxidative stress by activating the Nrf2/ARE pathway and Nrf2/ARE-related pathways, demonstrating antioxidant effects that help protect dopaminergic neurons from degeneration (Huang and Hsieh, 2021). Glutamic acid (Glu) is the predominant excitatory amino acid in the central nervous system, widely utilized. While Glu is not inherently toxic, it can modulate dopamine activity and release by activating specific Glu receptors in dopaminergic neurons, leading to potential toxicity. Research indicates that electroacupuncture can effectively modulate glutamate receptors, thereby decreasing glutamate levels in the striatum and cortex of mice (Jia et al., 2017). Damage to the substantia nigra area in PD is closely associated with an inflammatory response. Research indicates that the impact of acupuncture on improving motor function and preserving dopaminergic neurons may be linked to its ability to regulate intestinal microbial dysbiosis, consequently reducing neuroinflammation in PD mice (Jang et al., 2020). Based on current research conclusions, acupuncture can improve PD by regulating oxidative stress, immune inflammation, neurotransmitters, and mitochondrial function.

### 4.4 Implications for further study

Based on the results of the evaluations, we made several recommendations for improvement in response to the shortcoming. For example, to further clarify the conclusions on the effectiveness and safety of acupuncture for improving PD, reviewers should pre-register or publish the study protocols to avoid any risk of bias and to ensure the rigor of the SR process. During literature screening, a detailed and comprehensive search strategy should be provided with a list and explanation of excluded literature to avoid publication bias. When analyzing the data, subgroup analyses should be performed based on interventions, demographic information, etc. In terms of quality of evidence, future RCTs should address methodological issues through rigorous trial design, rational assessment, and critical analysis, and researchers should follow basic guidelines for clinical trial reporting, such as Comprehensive Standards for Trial Reporting (CONSORT) and Standards for Reporting of Interventions in Clinical Trials of Acupuncture (STRICTA2010).

### 5 Strengths and limitations

SRs based on high quality randomized controlled trials (RCTs) are essential for clinical decision-making in evidence-based medicine. However, the proliferation of SRs in recent years has raised concerns about their overall quality. Numerous studies have been published recently demonstrating the positive effects of acupuncture on PD. We systematically assessed the methodological quality, RoB and quality of evidence of relevant SRs using AMSTAR 2, ROBIS and GRADE tools, respectively. Limitations of our overview: First, we can only provide a comprehensive description of all SRs. Differences in study design and acupuncture intervention details may have resulted in a higher RoB for SR, thereby reducing the quality of the evidence and methods. Second, it must be acknowledged that quality assessment remains a subjective process and individual reviewers may judge each factor differently, leading to possible differences in results. Although evaluated and reviewed by two independent reviewers, the results of our study may differ from those of the other reviewers. Third, due to resource limitations, although seven databases were searched, they only included studies published in English or Chinese. This may create language bias and may exclude relevant SRs published in other languages.

## 5 Conclusion

The overview indicates that acupuncture is promising as an adjuvant therapy in improving movement disorder, depression, and sleep disorders in PD. Due to methodological flaws and the heterogeneity of available studies, current evidence is limited and inconclusive. High-quality, rigorously designed RCT studies should be conducted in the future to verify the effectiveness and safety of acupuncture in treating PD, which is crucial to advancing clinical decision-making and the development of treatment guidelines.

## Data Availability

The original contributions presented in the study are included in the article/Supplementary material, further inquiries can be directed to the corresponding authors.
