# Supplementary material for: An overview of systematic reviews of acupuncture for Parkinson’s disease
Source: Front Neurosci. 2024 Aug 30;18:1415008. doi: 10.3389/fnins.2024.1415008 (PMC11392918; doi:10.3389/fnins.2024.1415008)
Supplement: Supplementary file 1 [file Table_1.doc]

***Supplementary Table 1:*** The search strategy for each database

| **PubMed** |
| --- |
| #1 "Parkinson Disease"[Mesh] |
| #2 "idiopathic parkinsons disease"[Title/Abstract] OR "parkinsons disease idiopathic"[Title/Abstract] OR "parkinson disease idiopathic"[Title/Abstract] OR "parkinsons disease"[Title/Abstract] OR "idiopathic parkinson disease"[Title/Abstract] OR "primary parkinsonism"[Title/Abstract] OR "parkinsonism primary"[Title/Abstract] OR "paralysis agitans"[Title/Abstract] OR "Parkinsonism"[Title/Abstract] |
| #3 #1 OR #2 |
| #4 "Meta-Analysis"[Publication Type] |
| #5 "meta analysis"[Title/Abstract] OR "meta analysis"[Title/Abstract] OR "systematic review"[Title/Abstract] OR "systematic assessment"[Title/Abstract] OR "system evaluation"[Title/Abstract] OR "systematic evaluation"[Title/Abstract] OR "meta analysis as topic"[MeSH Major Topic] |
| #6 #4 OR #5 |
| #7 "Acupuncture"[MeSH Terms] |
| #8 Acupuncture[Title/Abstract] OR Acupuncture Treatment*[Title/Abstract] OR Acupuncture Therapy[Title/Abstract] OR Acupuncture Therapies[Title/Abstract] OR Pharmacoacupuncture[Title/Abstract] OR Acupotomies[Title/Abstract] OR Pharmacopuncture[Title/Abstract] OR Dry-needling[Title/Abstract] OR Body-acupuncture[Title/Abstract] OR Electroacupuncture[Title/Abstract] OR Electro-acupuncture[Title/Abstract] OR Auricular Acupuncture[Title/Abstract] OR Warm Needle[Title/Abstract] |
| #9 #7 OR #8 |
| #10 #9 AND #6 AND #3 |
| **Embase** |
| #1 ‘meta analysis’/ exp OR ‘meta analysis (topic)’/ exp |
| #2 ‘meta analysis’: ti,ab OR ‘meta analyses’: ti,ab OR ‘meta-analysis’: ti,ab OR ‘meta-analyses’: ti,ab OR meta analysis:ti,ab OR meta analysis: ti,ab OR ‘met-analysis’: ti, ab OR meta analyses: ti, ab OR metanalyses: ti,ab OR ‘clinical trial overview’: ti, ab OR ‘clinical trial overviews’: ti,ab |
| #3 ‘systematic review’/ exp OR ‘systematic review (topic)’/ exp |
| #4 ‘systematic review’: ti,ab OR ‘systematic reviews’: ti, ab |
| #5 #1 OR #2 OR #3 OR #4 |
| #6 'Acupuncture'/exp |
| #7 'Acupuncture':ab,ti OR 'Acupuncture Treatment*':ab,ti OR 'Acupuncture Therapy':ab,ti OR 'Acupuncture Therapies':ab,ti OR 'Pharmacoacupuncture':ab,ti OR 'Acupotomy':ab,ti OR 'Acupotomies':ab,ti OR 'Pharmacopuncture':ab,ti OR 'Dry-needling':ab,ti OR 'Body-acupuncture':ab,ti OR 'Electroacupuncture':ab,ti OR 'Electro-acupuncture':ab,ti OR 'Auricular Acupuncture':ab,ti OR 'Warm Needle':ab,ti OR 'Meridian*':ab,ti OR 'Ching Lo':ab,ti OR 'Jing Luo':ab,ti OR 'Jing mai':ab,ti OR 'Jingluo':ab,ti OR 'Jingmai':ab,ti |
| #8 #6 OR #7 |
| #9 ‘Parkinson Disease’/ exp OR ‘Parkinson’s Disease’ / exp OR ‘Parkinsonism’ / exp |
| #10‘Parkinson Disease’ : ti,ab OR ‘Parkinson’s Disease’ : ti,ab OR ‘Parkinsonism’ : ti,ab |
| #11 #9 OR #10 |
| #12 #5 AND #8 AND #11 |
| **Web of Science** |
| #1 TS =“meta analysis” OR TS =“meta analyses” OR TS =“meta-analysis” OR TS =“meta-analyses” OR TS = “meta-Analysis” OR TS =“metanalysis”OR TS =“ metaanalyses”OR TS =“systematic review” OR TS =“systematic reviews” OR TS =“clinical trial overview” OR TS =“clinical trial overviews” |
| #2 TS=‘Acupuncture’ OR ‘Acupuncture Treatment*’ OR ‘Acupuncture Therapy’ OR ‘Acupuncture Therapies’ OR ‘Pharmacoacupuncture’ OR ‘Acupotomy’ OR ‘Acupotomies’ OR ‘Pharmacopuncture’ OR ‘Dry-needling’ OR ‘Body-acupuncture’ OR ‘Electroacupuncture’ OR ‘Electro-acupuncture’ OR ‘Auricular Acupuncture’ OR ‘Warm Needle’ OR ‘Meridian*’ OR ‘Ching Lo’ OR ‘Jing Luo’ OR ‘jing mai’ OR ‘Jingluo’ OR ‘jingmai’ |
| #3 TS =“Parkinson Disease” OR TS =“Parkinson’s Disease” OR TS =“Parkinsonism” |
| #4 #1 AND #2 AND #3 |
| **The Cochrane Library** |
| #1 MeSH descriptor: [Parkinson’s Disease] explode all trees |
| #2 (Parkinson’s Disease):ab,ti,kw OR (Parkinson Disease):ab,ti,kw |
| #3 #1OR#2 |
| #4 (meta analysis):ab,ti,kw OR (meta analyses):ab,ti,kw OR (meta-analysis):ab,ti,kw OR (meta-analyses):ab,ti,kw OR (meta-Analysis):ab,ti,kw OR (metanalysis):ab,ti,kw OR (metaanalyses):ab,ti,kw OR (systematic review):ab,ti,kw OR (systematic reviews):ab,ti,kw OR (clinical trial overview):ab,ti,kw OR (clinical trial overviews):ab,ti,kw |
| #5 "Acupuncture":ti,ab,kw or "Acupuncture Treatment*":ti,ab,kw or "Acupuncture Therapy":ti,ab,kw or "Acupuncture Therapies":ti,ab,kw or "Pharmacoacupuncture":ti,ab,kw or "Acupotomy":ti,ab,kw or "Acupotomies":ti,ab,kw or "Pharmacopuncture":ti,ab,kw or "Dry-needling":ti,ab,kw or "Body-acupuncture":ti,ab,kw or "Electroacupuncture":ti,ab,kw or "Electro-acupuncture":ti,ab,kw or "Auricular Acupuncture":ti,ab,kw or "Warm Needle":ti,ab,kw or "Meridian*":ti,ab,kw or "Ching Lo":ti,ab,kw or "Jing Luo":ti,ab,kw or "Jing mai":ti,ab,kw or "Jingluo":ti,ab,kw or "Jingmai":ti,ab,kw |
| #6 #3 AND #4 AND #5 |
| **China National Knowledge Infrastructure** |
| ( ( (主题=系统评价 或者 题名=系统评价 或者 v_subject=中英文扩展(系统评价) 或者 title=中英文扩展(系统评价)) 或者 (主题=meta分析 或者 题名=meta分析 或者 v_subject=中英文扩展(meta分析) 或者 title=中英文扩展(meta分析)) ) 或者 ( (主题=荟萃分析 或者 题名=荟萃分析 或者 v_subject=中英文扩展(荟萃分析) 或者 title=中英文扩展(荟萃分析)) 或者 (主题=元分析 或者 题名=元分析 或者 v_subject=中英文扩展(元分析) 或者 title=中英文扩展(元分析)) ) ) 并且 ( ( (主题=针灸 或者 题名=针灸 或者 v_subject=中英文扩展(针灸治疗) 或者 title=中英文扩展(针灸疗法)) 或者 (主题=针刺 或者 题名=针刺激 或者 v_subject=中英文扩展(针刺疗法) 或者 title=中英文扩展(头针)) ) 或者 ( (主题=头针 或者 题名=头针 或者 v_subject=中英文扩展(体针) 或者 title=中英文扩展(体针)) ) ) 并且 ( ( (主题=帕金森病 或者 题名=帕金森 或者 v_subject=中英文扩展(帕金森病) 或者 title=中英文扩展(帕金森氏病)) |
| **Wanfang Database** |
| 检索表达式（中英文扩展&主题词扩展）： 主题:(系统评价+系统综述+meta分析+荟萃分析+元分析)*主题:(针刺 + 针灸+耳针+体针+舌针+电针+温针灸+腹针+头针+ 穴位+经络+经脉)*主题:(帕金森病+帕金森症+帕金森患者+帕金森综合征) |
| **Chongqing VIP** |
| (U=帕金森病 OR U=帕金森患者 OR U=帕金森综合征) AND (U=针刺 OR U=针灸 OR U=耳针 OR U=体针 OR U=舌针 OR U=电针 OR U=温针灸 OR U=腹针 OR U=头针 OR U=穴位 OR U=经络 OR U=经脉) AND (U=荟萃分析 OR U=系统综述 OR U=系统评价 OR U=Meta分析) |
